# Cuticular Waxes and Cutin in *Terminalia catappa* Leaves from the Equatorial São Tomé and Príncipe Islands

**DOI:** 10.3390/molecules28176365

**Published:** 2023-08-31

**Authors:** Helena Pereira, Rita Simões, Isabel Miranda

**Affiliations:** Centro de Estudos Florestais (CEF), Laboratório Associado Terra, Instituto Superior de Agronomia, Universidade de Lisboa, Tapada da Ajuda, 1349-017 Lisboa, Portugal; ritafabiana@isa.utl.pt (R.S.); imiranda@isa.ulisboa.pt (I.M.)

**Keywords:** cuticle, tropical almond, extracts, triacontanol, triterpenes, popular medicine, *α*-amyrin, *β*-amyrin, germanicol, lupeol

## Abstract

This study presents for the first time an analysis of the content and chemical composition of the cuticular waxes and cutin in the leaves of the widespread and important tropical species *Terminalia catappa*. The leaves were collected in the equatorial Atlantic islands of São Tomé and Príncipe, in the Gulf of Guinea. The epicuticular and intracuticular waxes were determined via dichloromethane extraction and their chemical composition via GC-MS analysis, and the content and monomeric composition of cutin were determined after depolymerization via methanolysis. The leaves contained an epidermal cuticular coverage of 52.8 μg cm^−2^ of the cuticular waxes (1.4% of mass) and 63.3 μg cm^−2^ (1.5% of mass) of cutin. Cuticular waxes include mainly *n*-alkanols and fatty acids, with a substantial proportion of terpenes in the more easily solubilized fraction, and sterols in the more embedded waxes. Cutin is mostly constituted by C16 fatty acids and dihydroxyacids, also including aromatic monomers, suggesting a largely linear macromolecular arrangement. The high proportion of triacontanol, *α*-amyrin, *β*-amyrin, germanicol, and lupeol in the easily solubilized cuticular fraction may explain the bioactive properties attributed to the *T. catappa* leaves via the popular medicine, which allows us to consider them as a potential source for the extraction of these compounds.

## 1. Introduction

*Terminalia catappa* L. is a tropical tree species in the family Combretaceae that is native to the tropical regions of Asia and northern Australia but was widely spread by humans, and at present, is common in the tropical parts of the Americas and in Africa. It is commonly called the tropical almond or Indian almond, but the names sea almond, wild almond, and umbrella tree are also used. *T. catappa* is a resilient species used as ornamentals due to its large and striking leaves and graceful canopy, its protection against strong winds, and also for providing nuts for consumption and being a source for local medicine [1,2].

*T. catappa* L. is a medium to large deciduous tree that grows to about 25 m or above, with an upright, symmetrical crown and long horizontal branches, and a bole that can go up to 150 cm in diameter, often with buttresses up to 3 m in height [3,4]. It is a salt-tolerant species that is very commonly found in coastal and beach areas or on tidal riverbanks in many tropical regions. The tree has been widely planted as an ornamental and shade tree throughout the tropics, often becoming naturalized, but it also grows in a variety of environments, from humid tropical climates to dry conditions.

The leaves are commonly 15–25 cm long and 10–14 cm broad, simple, and petiolate, with a glossy dark green colour that turns pinkish-reddish or yellow-brown before shedding. The leaves' anatomical features have been reported as follows: stomata are absent on the upper surface and present on the lower surface; simple unicellular trichomes are on the lower surface; the upper epidermal cells are barrel-shaped and single layered; the underlying palisade mesophyll is one layered with cylindrical elongated and closely arranged cells with many chloroplasts; and the spongy mesophyll cells are irregularly shaped, with 3–4 layers of thin-walled parenchyma cells with intercellular spaces [5,6,7].

*T. catappa* is widely known as a source of medicinal products with pharmacological benefits for the treatment of various ailments, mostly using the leaves and bark, while the nutritional value and health benefits of the fruit and seed are also recognized [8,9,10]. The species has become an integral part of traditional medicine within its native range and has been adopted by local communities in the various expansion areas for their application against diverse ailments such as fever, dysentery, asthma, and rheumatisms, to name a few. *T. catappa* leaves have been researched regarding their chemical composition including flavonoids, tannins, alkaloids, steroids, and carbohydrates [11,12]. Numerous research works also addressed *T. catappa* extracts’ bioactivity, e.g., its antioxidant, antitoxic, antimicrobial, and antifungal properties [13,14,15,16,17]. A curious application of the Indian almond leaves is by aquarists who keep fish native to tannin-rich waters by placing them in aquaria to mimic the natural conditions of the species (http://www.indianalmondleaves.com/, accessed on 20 June 2023).

No reports have been published on the leaf cuticle of *T. catappa*, nor on the chemical characterization of its cuticular waxes and cutin. The leaf cuticle is an important boundary membrane established between the plant and the environment, as it is involved in the regulation of non-stomatal water loss and gas exchange, and also confers resistance to several biotic and abiotic stresses [18,19]. The cuticle is a layer covering the epidermal cells that consists of an insoluble polyester matrix named cutin, and cuticular waxes that are soluble in low-polarity solvents [20,21]. Cuticular waxes are composed of two types: the intracuticular waxes embedded in the cutin matrix, and the epicuticular waxes deposited above the cutin as an external layer. Cuticle thickness, proportion of cutin and cuticular waxes, and their chemical compositions show a high degree of variability between the leaves of different species while also playing a role in the interactions between the leaf’s surface and environment, and therefore, at the forefront of the potential responses to the adverse or changing conditions [22,23,24].

This study presents, for the first time, an analysis of the content and chemical composition of the cuticular waxes and cutin in the leaves of *Terminalia catappa*. The trees were collected in the equatorial Atlantic islands of São Tomé and Príncipe, in the Gulf of Guinea, at three locations. A methodology was developed to determine the proportion of epicuticular and intracuticular waxes and of their chemical composition, as determined via GC-MS analysis, as well as the content and monomeric composition of cutin after depolymerization via methanolysis. It is the aim to enlarge knowledge on the chemical diversity of leaf cuticles, and, specifically for *T. catappa*, to have a first insight into the leaf cuticle composition of this widespread and important tropical species.

## 2. Results and Discussion

### 2.1. Cuticular Waxes Content

The content in the cuticular waxes in *T. catappa* leaves was determined via dichloromethane (DCM) solubilization via immersion at ambient temperature with a short period to extract the most accessible waxes, here referred to as epicuticular waxes, and a longer period at a higher temperature to further solubilize the waxes, here named as intracuticular waxes. The terms should therefore be taken with caution, and a more accurate designation would refer to the DCM extracts of whole leaves under the given experimental conditions. However, for simplicity, and following the usual terminology in cuticle studies, the terms epicuticular and intracuticular waxes are used here. It is known that the DCM applied on whole leaves does not extract compounds from the internal leaf tissues [25].

The content of the epicuticular and intracuticular waxes in *T. catappa* adult leaves is given in Table 1. On average, cuticular waxes represent 1.4% of the dry leaves’ mass (the proportion of the epicuticular and intracuticular waxes was 52.9% and 47.1%, respectively). The between-site variation in the content of the cuticular waxes was high in relation to the epicuticular waxes (e.g., 75% coefficient of variation of the mean), while the intracuticular waxes’ content showed less differences. 

Regarding leaf coverage, i.e., the amount per unit surface area of the two sides of the leaves, cuticular waxes corresponded to 52.8 μg/cm^2^. There is a large range of values for the leaf coverage and ratio of epicuticular to intracuticular waxes in different species, with reports of much higher values than those found here for *T. catappa* or, on the contrary, to much lower values. For instance, *Quercus suber* leaves have a substantial cuticular wax layer of 154.3–235.1 μg cm^−2^ [21], *Quercus petraea* leaves have 101.5–134.5 μg cm^−2^ and *Fagus sylvatica* 30.7–55.2 μg cm^−2^ [26], *Quercus ilex* leaves 71 μg cm^−2^ [27], *Quercus robur* 59 μg cm^−2^ [28], and *Quercus polymorpha* 199.4 μg cm^−2^ [29]. The ratio of epicuticular to intracuticular waxes, that was 1.1 in *T. catappa*, also varies largely between species with the predominance either of epicuticular waxes or of intracuticular waxes. For instance, the ratio ranged from high ratio values of 6.9 (*Clusia flava*) to 4.6 (*Garcinia spicata*), 3.5 (*Schefflera elegantissima*), and 2.7 (*Citrus aurantium*) [23].

### 2.2. Cutin Content

The cutin content of *T. catappa* adult leaves is given in Table 1. On average, cutin amounted to 1.5% of the dry leaves, corresponding to a leaf coverage of 63.3 μg/cm^2^. The between-site variation in cutin content was small. 

Cutin content in *T. catappa* leaves is much lower than the 7.1% reported for *Quercus suber* leaves [30]. It is known that cutin content in leaves varies widely among plant species, and a large range of cutin leaf coverage values has been reported, e.g., 340.6 μg cm^−2^ in *Rhazya stricta* [31], 203.8 μg cm^−2^ in *Gossypium barbadense* and 170.7 μg cm^−2^ in *Gossypium hirsutum* [32], 33.2 μg cm^−2^ in *Hedera helix* and 41.5 μg cm^−2^ in *Prunus laurocerasus* [33], and between 3 and 5 μg cm^−2^ for different *Camelia* spp. [34]. 

### 2.3. Composition of Cuticular Waxes

The composition of the epicuticular and intracuticular waxes is given in Table 2 by chemical family and is detailed in Table 3 by compound. It is clear that there is a difference in chemical composition between epicuticular and intracuticular waxes regarding the proportion of long chain lipids and the relative amounts of specific chemical classes and compounds.

Epicuticular waxes have a striking content of terpenes (51.9%, mainly *α*-amyrin, *β*-amyrin and germanicol), with *n*-alkanols (mainly triacontanol) also representing 17.3%, while fatty acids only amount to 5.7%, hydroxyacids 2.4%, and diacids practically absent. Intracuticular waxes are dominated by *n*-alkanols (39.0%, also with triacontanol as the main compound) and also have a large proportion of sterols (25.2%), while terpenes correspond to 10.5% and fatty acids to 8.5%. 

Cuticular waxes include compounds from different chemical families, namely long-chain aliphatics such as fatty acids, aldehydes, primary and secondary alcohols, ketones, alkanes, and cyclic compounds, such as triterpenoids, tocopherols and phytosterols, and aromatic compounds [19,35,36,37]. There is a large diversity in the composition of cuticular waxes of different species. For instance, in *Quercus suber* leaves, the cuticular wax layer was composed predominantly of triterpenes and aliphatic compounds with 61–72% and 17–23%, respectively [21], while in *Quercus ilex*, the most abundant components were *n*-alkanoic acids (38%) and *n*-alkanols (43–54%), with small amounts of the triterpenoids *α*-amyrin and *β*-amyrin [38]; in *Quercus robur*, the dominating classes were alcohols (about 70%), fatty acids (20%), aldehydes (28%), and several triperpenoids (8%) [39]. In *Castanea sativa*, the cuticular waxes consisted of a homologous series of wax lipids (esters, aldehydes, primary alcohols, and fatty acids) and large amounts of triterpenoids (*α*- and *β*-amyrin and lupeol, while *Fagus sylvatica* contained only wax lipids, without any triterpenoids [40]. In *Eucalyptus camaldulensis* and *E. globulus*, the cyclic compounds constituted about 39% and 76%, respectively [41,42].

A comparison between the chemical composition of epicuticular and intracuticular waxes in *T. catappa* leaves (Table 2) shows that the epicuticular layer predominantly concentrated triterpenoids and alkanols which were the main long-chain aliphatic compounds, while the intracuticular layer accumulated more alkanols and fatty acids, along with a high content in sterols. This is contrary to reports from other species for which long-chain aliphatic compounds were predominantly concentrated in the epicuticular layer, while triterpenoids were found in the intracuticular layer [43,44]. Similar results were obtained for a short time DCM extraction of *Quercus suber* leaves for which 95.5% of the extract was composed of *n*-alkanols and longer extractions were needed to solubilize the terpenes [25]. 

The substantial presence of triterpenoids in the easily solubilized surface waxes of *T. catappa* leaves is in line with their application in traditional medicine. In fact, most of the compounds present in the cuticular waxes of *T. catappa* (Table 3) have biological activities, which may explain the beneficial medicinal use of the leaves’ extracts. Triacontanol, a C_30_ *n*-alkanol, is a plant growth regulator found in epicuticular waxes that is involved in overcoming the negative effects of salt stress [45] and that is known as a growth promotor when exogenously applied to a number of plants [46,47]. *α-* and *β*-amyrins are triterpenoids (only differing in the placement of the methyl group) that have pharmacological activities exhibiting anti-inflammatory, antidiabetic, and anticancer effects, and are used as precursors for the biosynthesis of valuable bioactive compounds or as a lead compound for drug development effective in diabetes and atherosclerosis [48,49,50,51,52]. Germanicol is a pentacyclic triterpenoid found in various plants that exhibits selective antiproliferative activity against two human colon cancer cells lines mediated via the induction of apoptosis and the suppression of cell migration [53]. Lupeol is also a pharmacologically active pentacyclic triterpenoid with several potential medicinal properties, like anticancer and anti-inflammatory activity. 

The high proportion of triacontanol, *α*-amyrin, *β*-amyrin, germanicol, and lupeol in the easily solubilized fraction of cuticular waxes may explain the bioactive properties attributed to *T. catappa* leaves by the popular medicine. It also allows us to consider the leaves as a potential source for these compounds by their targeted extraction.

### 2.4. Composition of Cutin

The composition of cutin is given in Table 4 by chemical family and is detailed in Table 5 by compound. Cutin composition is dominated by fatty acids (64.8% of the total compounds), mainly including hexadecanoic acid (48.8%), with a substantial amount of hydroxyacids (18.0%), mostly of 10,16-dihydroxyhexadecanoic acid (15.1%), and the presence of aromatics (4.8%), mainly of methyl *p*-coumarate (2.5%).

This composition is in line with the overall cutin composition, i.e., cutin being a glyceridic polyester of C16 and C18 hydroxyacids and alkanoic acids with bonding to phenolic acids, namely to *p*-coumaric acid [54,55,56]. The relative proportion of the monomeric classes varies among species. For instance, in *Quercus suber*, the cutin contains mostly *ω*-hydroxyacids 44.4%, fatty acids 20.7%, *α,ω*-diacids 6.5%, and aromatics 12.8% [57], while *ω*-hydroxyacids represented 78% of the cutin in *Prunus laurocerasus*, 70% in *Citrus aurantium* [58], and 34% in *Camelina sativa* [34]. The proportion of phenolics also varies, e.g., 27% and 16% for *Gossypium barbadense* and *G. hirsutum*, respectively [32], and 13% to 21% in *Camelina* spp. [34].

Cutin provides strength and rigidity to the epidermal layer, and its macromolecular structure is determined via its monomeric composition and the functional groups that allow the establishment of ester bonds, i.e., the carboxylic and hydroxyl groups. In *ω*-hydroxy acids, the majority of the terminal hydroxyl groups participate in the ester bonds, but only half of the secondary hydroxyl groups are esterified while the number of unesterified carboxyl groups is very small [20,54,59]. The *ω*-hydroxy acids, with only a single hydroxyl group at the chain’s end, can only contribute to the formation of linear chains, while the presence of mid-chain hydroxyl groups allows the formation of dendritic structures [54].

In the case of *T. catappa* (Table 5), the cutin is of a C16 type, and the high content of unsubstituted alkanoic acids in comparison with the mid-chain hydroxylated *ω*-hydroxy acids indicates a predominantly linear structure. 

### 2.5. Experimental Considerations

The determination of cuticular waxes was made using whole leaves via solubilization in dichloromethane, with care to avoid contamination by leaf internal lipids that may be extracted via solvent entering the leaves through surface lesions or cut areas created during leaf preparation. Due to the large size of the *T. catappa* leaves, an adaptation was made to the solubilization methodology that we adopted previously for *Quercus suber* leaves [21,60]. A large and shallow glass container was used where the leaves could be immersed flat, and solubilization was made first to the more easily accessible compounds at the leaf surface (here called epicuticular waxes) via a short 5 min immersion at ambient temperature, followed by a 3 h long immersion at a temperature slightly below the boiling point of the solvent (by 3–4 °C) that solubilized the more entrapped material (here called intracuticular waxes). However, the classification as epicuticular and intracuticular waxes, as used here, should be taken with caution since the results refer to the DCM solubles, respectively, by short-ambient and long-hot solubilization conditions.

Dichloromethane was used as solvent, as previously tested, avoiding more polar solvents such as chloroform or acetone that may have an enhanced entrance to the inner leaf tissues [25].

The subsequent determination of cutin requires a reactive step for its depolymerization and monomer solubilization that was made via methanolysis using the previously tested conditions [30]. Since small leaf pieces were required to fit into the reaction vessel, a previous exhaustive Soxhlet extraction with DCM was made to remove all soluble lipid components that could contaminate the cutin compositional analysis. The yield obtained for these leaf internal DCM solubles was 0.6% (Table 1) that contained (Table 6 and Table 7) mostly fatty acids (51.9%, mainly hexadecanoic acid), aromatics (16.0%, with methyl-coumarate and several phenolics), sterols (6.0%, mainly *β*-sitosterol), alkanols (4.5%), and sugars (3.9%).

## 3. Materials and Methods

### 3.1. Sampling

The leaves were collected from mature T*erminalia catappa* L. trees growing in three locations in the São Tomé and Principe islands: site 1, in São Tomé’s urban coast (0°12′58″ N, 6°35′40″ E); site 2, in the sand front of the islet Ilhéu das Rolas (0°0′1″ N, 6°31′18″ E); and site 3, in the sand beach Boi of Príncipe island (1°40′51″ N, 7°27′36″ E). The islands have a tropical climate with an annual average rainfall of 900 mm with one long rainy season from September to May, and a mean temperature between 22 °C and 26 °C. 

The adult leaves were collected from several branches around the canopy below a height of approximately 2 m, from three trees in each site. The sampling was carried out in April 2023, and only complete and undamaged green leaves were taken. Three leaves for each site were chemically analyzed, and the results should therefore be taken as exploratory given this limited sampling. Leaf area was measured for the leaves that were chemically analyzed. The detailed methodology based on the image analysis of the leaves was reported in [21].

### 3.2. Determination of Cuticular Waxes

The solvent used for the extraction of the cuticular waxes was dichloromethane (DCM) [25]. Only whole leaves without any fracture or damage to the blade were taken. Since the leaves are large and did not fit into the usual chemical lab vessels, a methodological adaptation was made to our previous experimental procedures by using a shallow rectangular glass vessel (30 cm × 20 cm × 5 cm) where the leaves were immersed flat in the solvent. The leaves were first carefully dried at 60 °C overnight and weighted. 

The epicuticular waxes were solubilized via a short 5 min immersion at ambient temperature (23 °C) in 500 mL of solvent. The solvent was taken, evaporated in a rotavapor, and kept for chemical analysis. The extracted leaves were dried at 60 °C overnight and weighted.

The intracuticular waxes were extracted from the same leaves with 500 mL of dichloromethane using the same glass vessel in a water bath at 36 °C during 3 h. The solvent was taken, concentrated, and kept for chemical analysis. The leaves were dried at 60 °C overnight.

With the adopted experimental procedure, the cuticular waxes were extracted from both surfaces of the leaves. The amount of solubilized cuticular material was determined and expressed on a dry weight basis (g/1000 g dry leaf mass) and on a leaf surface area (as μg/cm^2^) corresponding to a leaf surface coverage, with the area being the two-sided leaf surface area. The detailed methodology was already reported, showing that little amounts of internal lipids or polysaccharides were solubilized under these conditions [25].

### 3.3. Determination of Cutin Content

In order to perform the depolymerization of cutin via methanolysis, it was necessary to cut the leaves into smaller pieces that would fit into the reaction vessel. This was made via the manual shredding of the leaves into about 1 cm^2^ pieces. However, in order to avoid any contamination in the chemical analysis of cutin from the internal lipids that would be accessed and solubilized via the reaction solvent, a previous Soxhlet extraction with dichloromethane was made during 6 h. The solvent was evaporated and kept for analysis, and the leaf pieces were dried at 60 °C. The extracted material obtained in this step is here called leaf internal DCM solubles. 

The depolymerization of cutin was carried out via transesterification with a sodium methoxide (NaOMe)-catalyzed methanolysis applied to the extracted leaves. The protocol followed the methodology applied to the cork suberin depolymerization [61] that was previously adapted to the cutin determination in cork oak leaves [57].

A sample of approximately 1.5 g extracted leaves was refluxed during 3 h with 100 mL of a methanolic 3% NaOCH_3_ solution, filtrated, washed with CH_3_OH, refluxed again with 100 mL of CH_3_OH during 15 min, filtrated, and then the combined filtrates were acidified to pH 6 with 2 M H_2_SO_4_ and evaporated to dryness. The residue was suspended in 50 mL of water and extracted three times with dichloromethane (50 mL each). The combined dichloromethane extracts were dried over anhydrous Na_2_SO_4_ and evaporated to dryness.

Cutin was quantified by determining the mass loss via methanolysis after drying and weighing the leaves’ residue, and it was expressed in percentage form of the initial dry weight basis (g/1000 g dry leaf mass) and on a leaf surface area (as μg/cm^2^), corresponding to a leaf surface coverage, with the area being the two-sided leaf surface area.

### 3.4. Chemical Composition of Cuticular Extracts and of Cutin

The extracts of the epicuticular and intracuticular waxes, the leaf internal DCM solubles, and the cutin monomers obtained via transesterification of the extracted leaves were solubilized in dichloromethane and derivatized into silylated derivatives for GC-MS analysis.

Aliquots (5 mL) of the dichloromethane extracts were evaporated under N_2_ flow and dried overnight at ambient conditions under a vacuum. The samples were derivatized in 100 μL of pyridine with 100 μL of bis(trimethylsilyl) trifluoroacetamide at 60 °C for 30 min, in order to trimethylsilylate (TMS) the hydroxyl and carboxyl groups into ethers and esters, respectively. 

The derivatized samples were immediately injected in a GC-MS Agilent 5973 MSD with the following GC conditions: Zebron 7HG-G015-02 column (30 m, 0.25 mm; ID, 0.1 μm film thickness), flow of 1 mL/min, injector of 280 °C, oven temperature program; 100 °C (1 min); a rate of 8 °C/min up to 250 °C; a rate of 5 °C/min up to 300 °C (5 min); a rate of 5 °C/min up to 350 °C (5 min); and a rate of 10 °C/min up to 380 °C (5 min). The MS source was kept at 220 °C and the electron impact mass spectra (EIMS) was taken at 70 eV. The compounds were identified as TMS derivatives by comparing with a GC-MS spectral library (Wiley, NIST, Gaithersburg, MD, USA) and with the published data, as reported in [21]. A semi-quantitative approach to the chemical composition was taken by using the integration of the peak area in the GC-MS total ion chromatograms, where each peak was quantified in the area proportion of the total chromatogram area. Duplicate analyses were made (with the standard deviation below 5%).

## 4. Conclusions

The widespread tropical species *Terminalia catappa* has an epidermal cuticular coverage of 52.8 μg cm^−2^ of cuticular waxes and 63.3 μg cm^−2^ of cutin. Cuticular waxes include mainly *n*-alkanols and fatty acids, with a substantial proportion of terpenes in the more easily solubilized fraction, and sterols in the more embedded waxes.

Cutin is mostly constituted by C16 fatty acids and dihydroxyacids, also including aromatic monomers, suggesting a largely linear macromolecular arrangement.

The high proportion of compounds with bioactive properties such as triacontanol, *α*-amyrin, *β*-amyrin, germanicol, and lupeol in the easily solubilized cuticular fraction allows us to consider the *T. catappa* leaves as a potential source for their extraction.

## Figures and Tables

**Table 1:** Chemical characterization of the leaves of *Terminalia catappa* of adult trees in three sites in the islands of São Tomé and Príncipe, regarding epicuticular and intracuticular waxes and cutin, in mg/g of dry leaves and in μg/cm^2^ of leaf surface. The leaf internal dichloromethane (DCM) solubles are also reported. Mean and standard deviation (±std) are also included.

Cuticular Lipid Content	Site 1	Site 2	Site 3	Mean ± Std
Cuticular waxes				
Epicuticular waxes (mg/g)	13.3	4.9	3.3	7.2 ± 5.4
Epicuticular waxes (μg/cm^2^)	36.4	26.0	14.2	25.6 ± 11.1
Intracuticular waxes (mg/g)	5.0	7.3	6.8	6.4 ± 1.0
Intracuticular waxes (μg/cm^2^)	13.8	38.2	29.7	27.2 ± 12.4
Total (mg/g)	18.3	12.2	10.1	13.6 ± 3.5
Total (μg/cm^2^)	50.2	64.3	43.9	52.8 ± 10.4
Internal DCM solubles (mg/g)	3.8	6.7	8.1	6.2 ± 2.2
Cutin (mg/g)	12.4	15.7	16.9	15.0 ± 2.3
Cutin (μg/cm^2^)	33.9	83.3	72.7	63.3 ± 21.2

**Table 2:** Composition by chemical class of the cuticular waxes (epicuticular and intracuticular) of leaves of *Terminalia catappa* adult trees in three sites in the islands of São Tomé and Príncipe, as a percentage of the total peak areas in the GC-MS chromatogram. Mean and standard deviation (±std) are also included.

Chemical Class	Epicuticular Waxes			Mean ± Std	Intracuticular Waxes			Mean ± Std
	Site 1	Site 2	Site 3		Site 1	Site 2	Site 3	
*n*-Alkanols	23.5	18.0	10.3	17.3 ± 6.6	54.4	33.5	29.0	39.0 ± 13.6
*n*-Alkanes	2.3	11.2	0.1	4.5 ± 5.9	2.1	1.1	7.1	3.4 ± 3.2
Fatty acids	9.4	5.5	2.3	5.7 ± 3.6	5.3	10.6	9.5	8.5 ± 2.8
Diacids	2.5	0.0	0.0	0.8 ± 1.4	0.0	0.4	0.0	0.1 ± 0.2
Hydroxyacids	7.3	0.0	0.0	2.4 ± 4.2	0.1	0.3	0.3	0.2 ± 0.1
Aromatic compounds	0.7	1.3	1.8	1.3 ± 0.6	0.3	0.6	0.2	0.4 ± 0.2
Sterols	0.0	1.1	0.7	0.6 ± 0.46	25.2	24.6	25.7	25.2 ± 0.6
Terpenes	38.6	47.7	69.3	51.9 ± 15.8	6.7	13.0	11.9	10.5 ± 3.4
Sugars	2.8	0.2	0.0	1.0 ± 1.6	0.0	1.8	0.0	0.6 ± 1.0
Others	0.6	1.6	0.9	1.0 ± 0.4	1.5	4.4	2.2	2.7 ± 1.5
Total identified	88.1	86.7	85.3	86.7 ± 1.4	95.5	90.1	85.8	86.7 ± 1.4

**Table 3:** Composition of the cuticular waxes (epicuticular and intracuticular) of leaves of *Terminalia catappa* adult trees as the averages of three sites in the islands of São Tomé and Príncipe, as a percentage of the total peak areas in the GC-MS chromatogram. Only compounds above 1% of total peak area at least in one site are included. Mean and standard deviation (±std) are also included.

Compounds	Epicuticular Waxes			Mean ± Std	Intracuticular Waxes			Mean ± Std
	Site 1	Site 2	Site 3		Site 1	Site 2	Site 3	
Alcohols								
Octacosanol	0.8	1.4	0.3	0.8 ± 0.4	1.3	0.6	2.1	1.3 ± 0.6
Triacontanol	20.2	14.3	9.2	14.6 ± 4.5	47.3	25.2	21.5	31.4 ± 13.9
Dotriacontanol	1.4	3.4	0.3	1.3 ± 0.9	5.2	4.9	4.3	4.8 ± 0.4
Tetratriacontanol	0.0	0.0	0.5	0.2 ± 0.2	0.6	2.7	1.0	1.4 ± 0.9
Alkanes								
Octacosane	0.8	6.6	0.0	2.5 ± 3.0	0.7	0.0	3.4	1.4 ± 1.5
Triacontane	1.5	4.3	0.0	1.9 ± 1.8	1.3	1.1	3.7	2.1 ± 1.2
Fatty acids								
Hexadecanoic acid	3.3	1.1	1.0	1.8 ± 1.0	1.7	3.5	3.8	3.0 ± 0.9
9,12-Octadecadienoic acid	0.2	0.0	0.0	0.1 ± 0.1	0.3	1.4	0.5	0.7 ± 0.5
9-Octadecenoic acid	0.1	0.4	0.0	0.2 ± 0.2	0.3	1.5	1.3	1.0 ± 0.5
Octadecanoic acid	1.3	0.4	0.5	0.7 ± 0.4	0.3	0.6	1.0	0.6 ± 0.3
Dotriacontanoic acid	0.0	1.7	0.0	0.6 ± 0.8	0.8	1.5	0.3	0.9 ± 0.5
*α,ω*-Diacids								
9,10-Dihydroxyoctadecanedioic acid, dimethyl ester	1.9	0.0	0.0	0.6 ± 0.9	0.0	0.4	0.0	0.1 ± 0.2
*ω*-Hydroxy fatty acids								
9,10,18-Trihydroxyoctadecanoic acid, methyl ester	1.2	0.0	0.0	0.6 ± 0.9				
22-Hidroxydocosanoic, methyl ester	5.8	0.0	0.0	1.9 ± 3.3	0.1	0.2	0.3	0.2 ± 0.1
Steroids								
Stigmasterol	0.0	1.1	0.7	0.6 ± 0.5	2.2	2.0	3.5	2.5 ± 0.7
*β*-sitosterol					22.5	21.9	21.2	21.9 ± 0.5
Terpenes								
Phytol	0.0	0.5	0.1	0.2 ± 0.2	0.2	0.9	1.4	0.6 ± 0.5
Squalene					0.2	1.3	2.1	1.2 ± 0.8
*β*-Amyrone	2.4	0.3	0.3	1.0 ± 1.0				
*β*-Amyrin	7.9	7.4	14.5	9.9 ± 3.3	3.4	2.6	2.7	2.7 ± 0.6
Germanicol	6.4	10.4	0.0	5.6 ± 4.3				
*α*-Amyrin	17.2	11.2	31.7	20.0 ± 8.6	1.1	1.6	1.8	1.5 ± 0.3
Lupeol	4.1	4.7	9.4	6.0 ± 2.4				
Betulinol	0.3	5.7	4.0	3.3 ± 2.3				
Oleanolic acid	0.1	5.7	2.1	1.9 ± 1.8				
Betulinic acid	0.1	1.7	0.8	0.9 ± 0.6				
Ursolic acid					0.0	1.2	0.2	0.5 ± 0.5
Corosolic acid					0.4	3.4	1.6	1.8 ± 1.2
Phytyl octadecanoate (C18:0)					0.4	1.2	1.8	1.1 ± 0.6
Sugars								
*meso*-erythritol					0.0	1.8	0.0	0.6 ± 0.6
Ribitol	2.8	0.0	0.0	0.9 ± 1.3				
Others								
1(3*H*)-Isobenzofuranone, 3-methyl-3-(2,4,6-trimethoxyphenyl)-	0.1	1.1	0.6	0.6 ± 0.5	0.6	3.2	1.9	1.9 ± 1.3
Total identified compounds	80.3	83.7	84.0	82.7 ± 2.1	92.1	85.7	81.9	86.5 ± 5.2

**Table 4:** Composition by chemical class of cutin of leaves of *Terminalia catappa* adult trees in three sites in the islands of São Tomé and Príncipe, as percentage of the total peak areas in the GC-MS chromatogram. Mean and standard deviation (±std) are also included.

Chemical Class	Cutin			Mean ± Std
	Site 1	Site 2	Site 3	
*n*-Alkanols	0.4	1.0	0.8	0.7 ± 0.3
Fatty acids	72.5	65.7	56.3	64.8 ± 8.1
Diacids	1.7	0.5	2.0	1.4 ± 0.8
Hydroxyacids	9.5	16.3	28.3	18.0 ± 9.5
Aromatic compounds	4.1	6.5	3.9	4.8 ± 1.5
Others	2.3	2.1	1.9	2.1 ± 0.2
Total identified compounds	90.5	92.1	93.2	91.9 ± 1.4

**Table 5:** Composition of the cutin of leaves of *Terminalia catappa* adult trees as the averages of three sites in the islands of São Tomé and Príncipe, as a percentage of the total peak areas in the GC-MS chromatogram. Only compounds above 1% of total peak area at least in one site are represented. Mean and standard deviation (±std) are also included.

Chemical Class	Cutin			Mean ± Std
	Site 1	Site 2	Site 3	
Fatty acids				
Hexadecanoic acid	4.0	5.1	8.8	6.0 ± 2.5
9-Decenoic acid	3.1	4.9	2.6	3.5 ± 1.2
Hexadecanoic acid, methyl ester	57.6	50.4	38.5	48.8 ± 9.6
Octadecanoic acid, methyl ester	2.3	3.2	3.1	2.8 ± 0.5
EIcosanoic acid, methyl ester	1.2	0.5	1.0	0.9 ± 0.4
*ω*-Hydroxy fatty acids				
16-Hydroxyhexadecanoic acid	1.9	2.6	3.9	2.8 ± 1.0
10,16-Dihydroxyhexadecanoic acid	7.4	13.6	24.2	15.1 ± 8.5
Phenolics				
Benzoic acid	0.7	1.0	0.8	0.8 ± 0.2
Benzeneacetic acid	1.0	0.0	0.0	0.3 ± 0.6
Methyl *p*-coumarate	0.5	1.8	0.9	1.0 ± 0.7
Methyl *p*-coumarate (isomer)	0.9	2.5	1.3	1.5 ± 0.8
Methyl isoferulate	0.0	1.1	0.0	0.4 ± 0.6
Others				
2-Pentadecenone, 6,10,14-trimethyl-	1.4	0.0	0.0	0.5 ± 0.8
Methyl 14-methoxyhexadecanoate	1.0	2.1	1.8	1.6 ± 0.6
Total identified compounds	83.0	88.7	86.9	86.2 ± 2.9

**Table 6:** Composition by chemical class of the leaf internal dichloromethane (DCM) solubles after extraction of cuticular waxes (epicuticular and intracuticular) of leaves of *Terminalia catappa* adult trees in three sites in the islands of São Tomé and Príncipe, as a percentage of the total peak areas in the GC-MS chromatogram. Mean and standard deviation (±std) are also included.

Chemical Class	Leaf Internal DCM Solubles			Mean ± Std
	Site 1	Site 2	Site 3	
*n*-Alkanols	5.2	2.6	5.6	4.5 ± 1.6
*n*-Alkanes	0.8	0.3	2.4	1.2 ± 1.1
Fatty acids	53.1	50.1	52.4	51.9 ± 1.6
Diacids	1.2	1.4	1.6	1.4 ± 0.2
Aromatic compounds	23.2	16.3	8.4	16.0 ± 6.1
Sterols	8.2	3.5	6.4	6.0 ± 2.4
Terpenes	0.3	0.0	1.2	0.5 ± 0.5
Sugars	1.1	2.9	7.7	3.9 ± 2.8
Total identified compounds	93.1	78.3	83.8	85.1 ± 6.1

**Table 7:** Chemical composition of the leaf internal dichloromethane (DCM) solubles after extraction of cuticular waxes (epicuticular and intracuticular) of leaves of *Terminalia catappa* adult trees in three sites in the islands of São Tomé and Príncipe, as a percentage of the total peak areas in the GC-MS chromatogram. Only compounds above 1% of total peak area at least in one site are included. Mean and standard deviation (±std) are also included.

Compound	Leaf internal DCM Solubles			Mean ± Std
	Site 1	Site 2	Site 3	
Alchools				
1,2-Hexadecanediol	1.6	1.7	0.9	1.4 ± 0.4
Dodecanol	0.4	0.0	1.1	0.5 ± 0.5
Triacontanol	2.4	0.5	2.0	1.6 ± 1.0
Dotriacontanol	0.7	0.3	1.0	0.6 ± 0.4
Alkanes				
Octacosane	0.3	0.1	1.1	0.5 ± 0.5
Triacontane	0.5	0.2	1.4	0.7 ± 0.6
Fatty acids				
Hexanoic acid	1.9	0.0	0.0	0.6 ± 1.1
Pentanoic acid, 4-oxo	3.0.	5.2	4.5	4.3 ± 1.1
Nonanoic acid	1.0	2.5	2.3	1.9 ±0.8
Decanoic acid	1.0	1.7	0.4	1.0 ± 0.6
Dodecanoic acid	0.7	1.5	0.5	0.9 ± 0.5
Tetradecanoic acid	7.2	9.9	5.1	7.4 ± 2.4
Hexadecanoic acid	28.0	21.5	25.3	24.9 ± 3.3
9-Octadecenoic acid	3.1	3.2	6.5	4.2 ± 1.9
Octadecanoic acid	3.3	2.9	4.0	3.4 ± 0.5
Eicosanoic acid	1.4	0.8	1.5	1.2 ± 0.4
Diacids				
Octanedioc acid	1.2	1.4	0.8	1.1 ± 0.3
Phenolics				
Benzoic acid	3.2	2.3	2.3	2.6 ± 0.5
Benzeneacetic acid	1.4	0.9	0.8	1.0 ± 0.3
Syringol	4.6	4.6	1.3	3.5 ± 1.9
Vanillin	2.6	2.3	1.1	2.0 ± 0.8
Vanillin acid	1.9	1.5	2.1	1.9 ± 0.3
Methyl *p*-coumarate	9.5	4.7	0.8	5.0 ± 4.4
Steroids				
Stigmasterol	1.3	0.5	1.3	1.1 ± 0.4
*β*-sitosterol	6.8	2.9	5.1	4.9 ± 1.9
Sugars				
Erythro-penitol, 2-deoxy-1,3,4,5 tetrakis-o-trimethylsilyl	1.1	0.6	1.3	1.0 ± 0.4
*meso*-erythritol	0.0	1.8	4.9	2.2 ± 2.5
Ribitol	0.0	0.6	1.6	0.7 ± 0.8
Total identified compounds	90.1	76.0	80.9	82.4 ± 7.2

## Data Availability

The original research data are available from the authors.
